# Validation of the Residual Feed Intake Model in Brangus Heifers: Determination of the Optimal Days on Feed Interval to Estimate Dry Matter Intake and Average Daily Gain

**DOI:** 10.3390/ani14142044

**Published:** 2024-07-12

**Authors:** Lauren E. Mahler, Mary Kimberly Mullenix, Terry D. Brandebourg, Lisa A. Kriese-Anderson

**Affiliations:** Department of Animal Sciences, Auburn University, Auburn, AL 36849, USA

**Keywords:** residual feed intake, Brangus, heifer, dry matter intake, carcass merit, reproductive performance

## Abstract

**Simple Summary:**

With the threat of climate change, a growing world population, and the demand for agricultural products rising, it’s crucial to enhance feed efficiency for the sustainability of beef production. Utilizing crossbred animals such as Brangus cattle to combine the heat tolerance of Brahman with the carcass merit of Angus is one potential strategy to meet these challenges, especially in warm humid climate regions. Incorporating selection for low residual feed intake (RFI) into breeding programs is another method that can speed genetic improvement of feed efficiency and reduce feed costs. However, accurately identifying such low RFI, feed efficient cattle is a timely and costly process, and little is known concerning the appropriate test conditions for Brangus cattle. We aimed to optimize the duration of RFI testing for daily feed intake in Brangus heifers. Our findings indicate that a 56-day test period is sufficient for accurate measurements of feed intake, with minimal loss in accuracy compared to a 70-day test. This reduces upfront costs for genetic evaluation and enhances the rationale for adopting RFI in Brangus breeding programs. Our research contributes to the long-term sustainability of beef production, aligning with the growing global demand for agricultural products and the need for increased efficiency in beef production.

**Abstract:**

Brangus cattle are gaining popularity in the Southeast U.S. due to the desirable heat tolerance from their Brahman influence combined with the superior carcass merit aspects of Angus genetics. However, little is known about the optimal evaluation conditions for this hybrid breed when placed on test for Residual Feed Intake (RFI), a heritable measure of feed efficiency that allows improvement in performance without altering carcass traits. To address this, dry matter intake (DMI) was measured on Brangus heifers for 70-d to determine the optimal days on feed required to estimate feed intake and ADG and assess if inclusion of ultrasound measures of carcass merit into the model impact RFI rankings for this breed. The 56-d test period had a regression coefficient of 0.96 (*p* < 0.0001), R^2^ = 0.94, rp = 0.97 (*p* < 0.0001), and rs = 0.97 (*p* < 0.0001), indicating little change in rank of cattle for DMI compared to a 70-d test. ADG was the limiting factor in determining test duration. Based upon examining only heifers that calved, ultrasound backfat measures should be included in the RFI model to normalize for differences in heifer maturity. Results from this study indicate that a test duration of 56-d is sufficient to accurately estimate DMI in this population. This data indicates on-test duration can be shortened, enhancing the rate of genetic change by reducing cost and increasing the number of animals that can be tested annually.

## 1. Introduction

Feed requirements for maintenance are estimated to represent up to 75% of the total annual carrying costs for the beef cow herd in modern production systems [1]. The cost of feed is an unpredictable input due to market fluctuations and the influence of seasonality on growing seasons, making the reliance upon stored feed to meet feed requirements within the beef industry problematic [1]. Additionally, human population growth is dictating a need to increase food production while simultaneously increasing competition for cereal grains commonly used in animal rations [2,3]. Therefore, improving feed utilization and efficiency is important to enhance the sustainability of beef production and strengthen the nutritional security of humans.

Ideally, selection for improved feed efficiency would reduce feed inputs without compromising other economically relevant traits in seedstock like carcass merit or reproductive performance [4,5]. Unfortunately, using traditional measures of feed efficiency, such as feed conversion ratio (FCR), as selection criterion often have negative impacts such as undesirable increases in mature size, maintenance requirements, and dry matter intake (DMI) [4,5,6,7]. On the other hand, residual feed intake (RFI) is a heritable measure of feed efficiency that is phenotypically independent of growth rate and body weight [4,6,8,9,10]. Defined as the difference between observed and predicted dry matter intake necessary to meet growth and maintenance energy requirements with the latter calculated from regression of feed intake on gain and metabolic midweight (MMWT), RFI is independent of the level of animal production [8,11]. Thus, incorporating RFI as a selection criterion into cattle breeding programs allows continual improvements in herd-wise feed efficiency with fewer antagonistic selection effects on the phenotype of progeny. It is estimated that the incorporation of RFI into selection programs could improve profitability for beef producers by as much as 33% due to the resulting decreases in daily DMI observed for a given rate of gain exhibited by progeny [12,13].

Brangus cattle are a breed of importance in the Southeast U.S. due to their adaptability, growth efficiency, and reproductive performance. The breed’s origins can be traced back to mid-20th century crossbreeding programs seeking to combine the hardiness and heat tolerance of Brahman cattle with the superior meat quality of Angus [14]. Importantly, Brangus cattle are adaptive to grazing-based systems and exhibit desirable reproductive performance, calving ease, and maternal instincts [15,16,17]. Combined, these phenotypic traits position Brangus as a candidate breed for further genetic improvement of feed efficiency. According to the Beef Improvement Federation (BIF) Guidelines, a 42-day testing period is indicated to accurately measure daily feed intake while on-test durations of at least 56-days are necessary for gain tests effectively setting the duration for RFI trials at no less than 56-days [18]. However, only studies using a test period of 70-d for Brangus cattle currently exist in the literature [19,20,21,22,23]. It is unclear if hybrid breeds such as Brangus cattle conform to testing requirements that have largely been informed by trials utilizing composite breeds without *Bos indicus* influence. Reducing the time Brangus cattle are at centralized testing facilities to determine their RFI status would reduce the upfront costs associated with genetic evaluation and allow more cattle to be tested annually, which would have a significant financial impact on Brangus producers.

BIF Guidelines currently recommend a RFI model computed by the regression of DMI on ADG, calculated by linear regression, and MMWT. While RFI is phenotypically independent of body weight and gain, some studies have found weak correlations between RFI and carcass traits such as adjusted backfat thickness [17,24,25,26,27,28,29]. One study that investigated this relationship in Brangus heifers reported a moderate genetic correlation between RFI and final ultrasound backfat thickness, suggesting that reduced subcutaneous fat is associated with lower RFI [29]. To eliminate any potential antagonistic impact that genetic selection for RFI may have on heifer development, several studies have suggested that ultrasound measures of body composition should be included in the RFI model [17,29,30]. However, the relationship between ultrasonically measured backfat depth and RFI status is currently unsettled [31,32]. Additionally, sound reproductive performance is essential in maintaining a profitable cattle operation, and some initial short-term studies indicate selection for favorable RFI may cause a later calving day [33,34,35].

Given there are no reports in the literature where current BIF guidelines for RFI were used for Brangus heifers on-test and the need to include ultrasound 12th rib fat depth in the RFI model is unresolved, the objectives of this study were to (1) determine the optimal days on feed for accurately measuring dry matter intake and average daily gain in Brangus heifers and (2) assess if ultrasound measures of carcass merit increase feed intake model accuracy. Here it is hypothesized that if optimal test duration for Brangus heifers is like that established for composite breeds then optimal test duration in the present study will reflect a 56-d interval. Furthermore, if inclusion of ultrasound backfat in the RFI model improves selection accuracy then adjusted RFI models will account for significantly more variation in DMI than unadjusted models.

## 2. Materials and Methods

### 2.1. Animals and Design

All procedures involving animals were approved by the Auburn University Animal Care and Use Committee (IACUCC 2014-2483). To determine the optimal days on feed interval necessary to estimate dry matter intake and average daily gain for Brangus heifers, 70-d residual feed intake trials were conducted using the Calan gate system to measure individual animal daily intake according to previous studies and BIF guidelines [18,36,37]. Average daily gain was measured by weighing animals biweekly. These data were then utilized to determine RFI, and rank individuals based upon the models described below. Animal inclusion and test rations strictly conformed to BIF guidelines for test birth dates, body weights, and ration energy requirements [18]. Heifers were returned to producer farms following test competition, where they were bred and reproductive outcomes recorded.

Daily feed intake was measured on 186 Brangus replacement heifers obtained from two purebred southeastern Brangus breeders. Angus and Brahman parentage was determined from producer lineage and breeding records. A total of 186 heifers were delivered to the Auburn University Beef Cattle Evaluation Center (AUBCE) during 2014 and 2015. Seven contemporary groups were assigned on testing to determine daily feed intake based on date of trial and farm of origin (Table 1).

The Auburn University Beef Cattle Evaluation Center has 8 pens, each fitted with 12 Calan^®^ gates (American Calan, Northwood, NH, USA). Each pen of cattle had indoor and outdoor access with a capacity of 12 cattle per pen. Pens were 6.1 by 9.1 m inside and 18.3 by 92.7 m outside. The outside portion of each pen was 18.6 m at the widest point by 92.7 m long and divided into three 6.2-m strips representing 575 m^2^ total or 48 m^2^/heifer. Paddocks contained common bermudagrass (*Cynodon dactylon* L.) as the forage base though paddocks were grazed down before initiation of test. Paddock size was insufficient to impact estimates of dry matter intake for the 12 animals that shared free access to a given paddock [36,37]. The on-test dates for each contemporary group are indicated in Table 1. Heifers were allowed access to a different strip of forage weekly, which served to minimize erosion and promote hoof health. Heifers had continuous access to automatic water troughs.

Heifers were transported to the AUBEC on 18-wheeler cattle trucks from their farm of origin. Heifers were randomly unloaded into one of the eight pens. Upon arrival, heifers were allowed to rest a minimum of 8 h prior to processing. Heifers were given access to hay and water. At processing, heifers were weighed and measured for hip height. Heifers were then placed in pens based on hip height and weight to minimize social hierarchy effects.

Heifers were trained to their individual Calan^®^ gates during a 21-d acclimation period. Initially, gates remained open and heifers were group fed the diet described in Table 2. The diet was formulated to be 2.4 Mcal/NE_m_ while meeting daily nutrient requirements for growing heifers as indicated by the Nutrient Research Council for Beef Cattle. Each pen was initially offered 2% body weight (BW) of the diet. Researchers observed and recorded heifers eating from each gate each day during the acclimation phase. Once the majority of heifers were observed eating, Calan^®^ gates were locked and heifers were fitted with transponders. The gate each heifer was assigned was determined by the observation data. Not all heifers could be trained to the Calan^®^ gates. Heifers unable to open their gate were excluded from the study.

Following the adaptation period, heifers underwent a 70-d feed intake trial to measure daily feed intake and growth performance. Heifers were fed twice a day to target *ad libitum* amounts such that 0.45 kg to 0.91 kg of feed were left in their bunks at each feeding. Orts were weighed each morning. Heifers were weighed on-test two consecutive days, designated as d-1 and d 0. Heifers were weighed and measured for hip height every 14-d. At the conclusion of 70-d, each heifer was weighed off-test on 2 consecutive days. Carcass ultrasound measurements of 12th rib fat, longissimus dorsi area, and percent intramuscular fat were taken by a certified ultrasound technician within 7 d of test completion. Ultrasound data were collected by an Ultrasound Guidelines Council certified technician using an Aloka 500 (Aloka America, Wallingford, CT, USA) with a 17-cm transducer using Centralized Ultrasound Processing to interpret scans (Ames, Iowa). Upon completion of each trial, health checks were performed by a veterinarian and heifers were transported via 18-wheeler cattle trucks to their respective farms whereby they were housed in pastures that prevented none-to-nose contact with animals in other pastures for 28-d before heifers were finally reintroduced into resident herds. Each farm was responsible for the breeding and calving of heifers.

### 2.2. Criteria for Heifer Inclusion

Data were edited for incomplete feed records and heifer age. According to BIF Guidelines, heifers must be at least 240 d at the initiation of the feed trial and no older than 390 d at the completion of the feed trial [18]. A total of 79 heifers were removed from the data analysis that did not fall within the recommended age range according to BIF Guidelines, leaving 186 records for this study. Individual feed intake was also checked to ensure total intake was within ±4 SD of their contemporary group. 

### 2.3. Statistical Analysis

ADG can be determined by two methods. Individual animal average daily gain (ADG_1_) was computed by the linear regression of weight on day of test using the PROC REG procedure in SAS (version 9.4, SAS Inst. Inc., Cary, NC, USA). ADG_1_ was derived from the following linear regression equation:

Y_i_ = β_0_ + β_1_X_i_ + e_i_

where:

Y_i_ = weight of animal at observation i

β_0_ = Y-intercept (initial BW)

β_1_ = regression coefficient (ADG_1_)

X_i_ = days on test at observation i

e_i_ = error in weight at observation i

ADG_2_ is derived from the following equation:

ADG_2_ = (Final BW − Initial BW)/days on test

Metabolic midweight (MMWT) was derived using both ADG_1_ and ADG_2_, resulting in the following:

MMWT_1_ = (Final BW − (0.5 × days on test × ADG_1_))^0.75^

MMWT_2_ = (Final BW − (0.5 × days on test × ADG_2_))^0.75^

Residual feed intake (RFI) was calculated as actual dry matter intake (DMI) minus expected DMI to meet growth and maintenance energy requirements [22]. It is assumed RFI is normally distributed with a mean of zero. Expected DMI is derived through a base model:

Y_i_ = β_0_ + β_1_ADG + β_2_MMWT + e_i_

where:

Y_i_ = expected DMI

β_0_ = regression intercept

β_1_ = partial regression coefficient of DMI on ADG

β_2_ = partial regression coefficient of DMI on MMWT

e_i_ = RFI

Additionally, RFI was determined by adjusting for 70 d ultrasound 12th rib fat (UBF) depth (RFI_bf_). The model adjusted for 12th rib fat depth for RFI used:

Y_i_ = β_0_ + β_1_ADG + β_2_MMWT + β_3_UBF + e_i_

where:

Y_i_ = expected DMI

β_0_ = regression intercept

β_1_ = partial regression coefficient of DMI on ADG

β_2_ = partial regression coefficient of DMI on MMWT

β_3_ = partial regression coefficient of DMI on UBF

e_i_ = RFI_bf_

All RFI values were derived using the PROC GLM procedure in SAS (version 9.4, SAS Inst. Inc., Cary, NC). A maximum of four RFI values were determined for each individual heifer by the following equations:

Model 1: Y_i_ = β_0_ + β_1_ADG_1_ + β_2_MMWT_1_ + e_1_

Model 2: Y_i_ = β_0_ + β_1_ADG_1_ + β_2_MMWT_1_ + β_3_UBF + e_2_

Model 3: Y_i_ = β_0_ + β_4_ADG_2_ + β_5_MMWT_2_ + e_3_

Model 4: Y_i_ = β_0_ + β_4_ADG_2_ + β_5_MMWT_2_ + β_3_UBF + e_4_

where:

Y_i_ = expected DMI

β_0_ = regression intercept

β_1_ = partial regression coefficient of DMI on ADG_1_

β_2_ = partial regression coefficient of DMI on MMWT_1_

β_3_ = partial regression coefficient of DMI on UBF

β_4_ = partial regression coefficient of DMI on ADG_2_

β_5_ = partial regression coefficient of DMI on MMWT_2_

e_i_ = RFI_1_

e_2_ = RFI_bf1_

e_3_ = RFI_2_

e_4_ = RFI_bf2_

Once RFI values were determined for heifers using each model, heifers were classified into one of three categories. Heifers classified as high, or inefficient, RFI heifers were more than 1 SD above the mean within the contemporary group. Heifers classified as low, or efficient, RFI heifers were more than 1 SD below the mean within the contemporary group. Heifers within 1 SD of the contemporary group were classified as medium, or average, RFI heifers. Heifers received an RFI classification for each model.

The PROC REG procedure in SAS (version 9.4, SAS Inst. Inc., Cary, NC, USA) was used to regress RFI_1_ on RFI_bf1_, RFI_2_ on RFI_bf2_, RFI_1_ on RFI_2_, and RFI_bf1_ on RFI_bf2_ to estimate the linear relationship between the models. The PROC CORR procedure in SAS was used to determine Pearson and Spearman correlations among the four models. Measures of agreement were determined between RFI_1_ and RFI_bf1_, RFI_2_ and RFI_bf2_, RFI_bf1_ and RFI_bf2_, and RFI_1_ and RFI_2_ using the PROC FREQ procedure in SAS. The AGREE option in the TABLE statement provided the respective kappa coefficient, standard error, and 95% confidence limits. The TEST WTKAP option within the PROC FREQ procedure computes the hypothesis test for weighted kappa values, where H_0_ = 0. Kappa values were used to determine the level of agreement between each RFI model pair, where <0.4 = low level of agreement beyond chance, 0.40–0.75 = fair to good level of agreement beyond chance, and >0.75 = high level of agreement beyond chance.

***Test Length:*** To assess whether a shorter feeding period could be implemented to accurately determine feed intake and ADG, subsets of the 70-d trials were created comparing on-test durations of 14, 28, 42, and 56-d. For each on-test duration, expected feed intake model components were estimated using both ADG_1_, ADG_2_ and MMWT_1_, MMWT_2_ definitions. The PROC REG procedure in SAS was then used to regress RFI, DMI, ADG, and MMWT for the full test (d 0 to 70) on the RFI, DMI, ADG, and MMWT values from the shorter tests. The CORR procedure in SAS was used to determine Pearson correlations for average DMI, RFI, ADG, and MMWT values, as calculated above, from a full 70-d test to these values from shorter on-test durations. Spearman rank correlations were also calculated to investigate potential changes in animal rank for d 70 average DMI, RFI, ADG, and MMWT when compared to the shorter testing periods. The relationship between ADG_1_ and ADG_2_ was further investigated to determine the best indicator of 70-d ADG using the PROC REG procedure in SAS to regress ADG_1_ values on ADG_2_ values for the 56-d and 70-d test. The CORR procedure in SAS was used to determine Pearson and Spearman correlations between ADG_1_ and ADG_2_ for 56 d and between ADG_1_ and ADG_2_ for 70-d. No ultrasound carcass data were included in these analyses since ultrasound data were only collected at the conclusion of the 70-d test.

***Effects of RFI on measures of growth:*** Independent variables of RFI classification, farm, sire, and trial were used in a general linear model to assess their impact on initial BW, final BW, DMI, ADG, MMWT, and UBF. Heifers without sire records were omitted from this analysis. Calving records were obtained on 54 heifers from trials conducted beginning in June and December of 2014. Independent variables included farm, classification, and sex of calf and were used in a general linear model to assess their impact on age at first calving for the four models. Calving age of each heifer was determined as calving date minus date of birth. The PROC GLM procedure of SAS was used for these analyses. Least squares means was used to separate means with a significant *p*-value set at 0.05. Further analysis between age at first calving and off-test BW were performed using the PROC CORR and PROC REG procedure of SAS.

## 3. Results

### 3.1. Growth Performance on Test

Simple means for performance traits by contemporary group indicated no differences (*p* > 0.05) in body weight or ADG between contemporary groups while on test (Table 3). RFI as adjusted for ADG (determined by linear regression) and metabolic midweight was calculated for each individual heifer following 70 days on test. All heifers were then classified as low (LRFI), medium, or high (HRFI) based on their RFI values. Low, medium, and high group RFI means were –1.44, −0.08, and 1.43, respectively, with low and high RFI group means separated by greater than two standard deviations (*p* < 0.0001) and *r*^2^ = 0.58 for DMI regressed on ADG and mid weight. As anticipated, there were no differences in initial BW (*p* < 0.82), final BW (*p* < 0.82), or ADG (*p* < 0.96) between LRFI, average, and HRFI groups (Table 4). Importantly, however, DMI differed significantly across groups with DMI lowest in LRFI heifers and highest in HRFI heifer (*p* < 0.0001) as HRFI heifers consumed 33% more feed/day to achieve similar gain as LRFI heifers. There was a tendency for UBF to be lower in LRFI heifers compared to average and HRFI groups (*p* = 0.10).

### 3.2. Determination of the Optimal Days on Feed for Accurately Measuring DMI and ADG in Brangus Heifers

The results of regressing 70-d DMI on shorter test durations are shown in Table 5. Regression coefficients increased with increasing test period duration maximizing at 0.96 (*p* < 0.0001) for a 56-d test. The 56-d test period had a *R*^2^ of 0.94, a Pearson correlation coefficient of 0.97 (*p* < 0.0001), and a Spearman correlation coefficient of 0.97 (*p* < 0.0001), indicating little change in rank of cattle for DMI compared to a 70-d test. Results for the 42-d duration were like those seen at 56 d. However, regression coefficients and correlations associated with regressing 70-d DMI on the shorter test lengths of 14 d or 28 d were less strong and associated with substantial differences in individual animal RFI ranking.

Results from the regression of ADG_1_ and ADG_2_ from the 70-d test on shorter test durations are shown in Table 6. Results from the regression of ADG_1_ values from the 70-d test on shorter test durations indicate regression coefficients increased as test period duration approached the 70-d benchmark, maximizing at 0.84 (*p* < 0.0001) for a 56-d test. The 56-d test period had a *R*^2^ of 0.86, a Pearson correlation coefficient of 0.93 (*p* < 0.005), and a Spearman correlation coefficient of 0.90 (*p* < 0.0001). Likewise, results from the regression of ADG_2_ values from the 70-d test on shorter test durations indicate regression coefficients increased as test period duration approached the 70-d benchmark, maximizing at 0.84 (*p* < 0.05) for a 56-d test. ADG_2_ for the 56-d test period had a *R*^2^ of 0.74, a Pearson correlation coefficient of 0.86 (*p* < 0.05), and a Spearman correlation coefficient of 0.86 (*p* < 0.005).

Linear regression of ADG_1_ values on ADG_2_ values for 56-d and 70-d confirmed the two measures are similar. Regression coefficients maximized at 1.06 (*p* < 0.0001) for the 56-d test and decreased slightly for the 70-d test to 0.99 (*p* < 0.0001). The 56-d test period had an *R*^2^ of 0.92, a Pearson correlation coefficient of 0.96 (*p* < 0.0001), and a Spearman correlation coefficient of 0.95 (*p* < 0.0001). The 70-d test period had an *R*^2^ of 0.93, a Pearson correlation coefficient of 0.97 (*p* < 0.0001), and a Spearman correlation coefficient of 0.96 (*p* < 0.0001), indicating few rank changes of cattle regardless of how ADG was calculated.

Results from the regression of RFI_1_ and RFI_2_ from the 70-d test on shorter test durations are shown in Table 7. Results from the regression of ADG_1_ values from the 70-d test on shorter test durations indicate regression coefficients increased as test period duration approached the 70-d benchmark, maximizing at 0.93 (*p* < 0.0001) for a 56-d test. The 56-d test period had a *R*^2^ of 0.90, a Pearson correlation coefficient of 0.95 (*p* < 0.005), and a Spearman correlation coefficient of 0.995 (*p* < 0.0001). Likewise, results from the regression of RFI_2_ values from the 70-d test on shorter test durations indicate regression coefficients increased as test period duration approached the 70-d benchmark, maximizing at 0.91 (*p* < 0.05) for a 56-d test. RFI_2_ for the 56-d test period had a *R*^2^ of 0.88, a Pearson correlation coefficient of 0.94 (*p* < 0.01), and a Spearman correlation coefficient of 0.93 (*p* < 0.001).

### 3.3. Assessment of the Impact of Including Ultrasound Measures of Carcass Merit on RFI Model Accuracy

Four RFI equations (Model 1: unadjusted RFI_1_; Model 2: RFI_BF1_; Model 3: unadjusted RFI_2_; Model 4: RFI_BF2_) were utilized to determine four separate RFI values for each individual heifer. These RFI models were then compared to determine which best accounted for variations in DMI, ADG, and MMWT.

When unadjusted models were compared to one another, Model 1 (RFI_1_, n = 186) accounted for 0.49 of the variation in DMI explained by ADG_1_ and MMWT_1_. Model 3 (RFI_2_, n = 186) accounted for 0.50 of the variation in DMI explained by ADG_2_. Pearson and Spearman correlation coefficients of 1.00 (*p* < 0.0001) and 0.99 (*p* < 0.0001) between RFI_1_ and RFI_2_, respectively, indicate they are nearly identical with little reranking of individuals with respect to determining RFI values. Linear regression of RFI_1_ on RFI_2_ revealed a regression coefficient of 1.00 ± 0.01 (*p* < 0.0001), which did not differ from 1 (95% confidence limits; 0.98 < β < 1.01) indicating model equivalency.

Next, unadjusted RFI models were compared to the adjusted versions, which included measures of backfat. When comparing Model 1 (RFI_1_, n = 186) and Model 2 (RFI_bf1_, n = 176), Model 2 accounted for an additional 2% of the variation in DMI with a *R*^2^ of 0.51. Pearson and Spearman correlation coefficients between RFI_1_ and RFI_bf1_ were 0.91 (*p* < 0.0001) and 0.89 (*p* < 0.0001), respectively. Out of 176 heifers with backfat records, 28 changed ranks. The following rank changes occurred; high to medium (*n* = 7), medium to low (*n* = 8), medium to high (*n* = 4), and low to medium (*n* = 9). The reranking of individuals for RFI was minimal despite the inclusion of ultrasound backfat records appearing to explain more variation within the model.

Comparing Model 3 (RFI_2_) and Model 4 (RFI_bf2_), the inclusion of backfat thickness allowed Model 4 (RFI_bf2_) to account for an additional 2% of the variation in DMI with a *R*^2^ of 0.52. RFI_2_ and RFI_bf2_ had strong Pearson and Spearman correlation coefficients of 0.93 (*p* < 0.0001) and 0.90 (*p* < 0.0001), respectively. Out of 176 heifers with backfat records, 24 changed ranks. The following rank changes occurred; high to medium (*n* = 2), medium to high (*n* = 9), low to medium (*n* = 7), and medium to low (*n* = 6). Linear regression of RFI_2_ on RFI_bf2_ revealed a regression coefficient of 1.00 ± 0.03 (*p* < 0.0001), which did not differ from 1 (95% confidence limits; 0.94 < β < 1.06).

RFI_bf1_ and RFI_bf2_ had strong Pearson and Spearman correlation coefficients of 0.96 (*p* < 0.0001) and 0.95 (*p* < 0.0001), respectively. Linear regression of RFI_bf1_ on RFI_bf2_ revealed a regression coefficient of 1.00 ± 0.02 (*p* < 0.0001), which did not differ from 1 (95% confidence limits; 0.96 < β < 1.04).

Consistent with the strength of relationship between models demonstrated by correlation analysis, measures of association show similar strength of agreement between models. The kappa coefficient between RFI_1_ and RFI_2_ was 0.84 (95% confidence limits; 0.77 < β < 0.92), which indicates high agreement. The hypothesis test confirms rejection of the null hypothesis of no agreement for all the models, suggesting the true kappa is greater than zero. The kappa coefficients between RFI_bf1_ and RFI_bf2_, RFI_2_ and RFI_bf2_, and RFI_1_ and RFI_bf1_ have the values 0.75 (95% confidence limits; 0.65 < β < 0.84), 0.75 (95% confidence limits; 0.65 < β < 0.84), and 0.67 (95% confidence limits; 0.56 < β < 0.79), respectively, indicating fair to good levels of agreement.

Next, to verify that these relationships hold for heifers with a positive reproductive outcome, calving records were analyzed from contemporary groups 3 and 7. The four models were evaluated against a subset of the data that included only heifers that calved (n = 54). Model 1 (RFI_1_) accounted for 0.43 of the variation in DMI explained by ADG_1_ and MMWT_1_. Model 2 (RFI_bf1_) accounted for an additional 4% of the variation in DMI due to the inclusion of 70 d ultrasound backfat thickness measures into the model (RFI_bf1_) with a *R*^2^ of 0.47. RFI_1_ and RFI_bf1_ had Pearson and Spearman correlation coefficients of 0.90 (*p* < 0.0001) and 0.90 (*p* < 0.0001), respectively. Linear regression of RFI_1_ on RFI_bf1_ revealed a regression coefficient of 0.88 ± 0.06 (*p* < 0.0001), which did not differ from 1 (95% confidence limits; 0.76 < β < 1.00). Model 3 (RFI_2_) accounted for 0.44 of the variation in DMI explained by ADG_2_ and MMWT_2_. Model 4 (RFI_bf2_) accounted for an additional 4% of the variation in DMI due to the inclusion of 70 d ultrasound backfat thickness measures into the model (RFI_bf2_) with a *R*^2^ of 0.48. RFI_2_ and RFI_bf2_ had strong Pearson and Spearman correlation coefficients of 0.94 (*p* < 0.0001) and 0.95 (*p* < 0.0001), respectively. Linear regression of RFI_2_ on RFI_bf2_ revealed a regression coefficient of 1.02 ± 0.05 (*p* < 0.0001), which did not differ from 1 (95% confidence limits; 0.92 < β < 1.11).

When comparing the two unadjusted models, RFI_1_ and RFI_2_ had the strongest Pearson and Spearman correlation coefficients at 1.00 (*p* < 0.0001) and 0.99 (*p* < 0.0001), respectively. Linear regression of RFI_1_ on RFI_2_ revealed a regression coefficient of 1.00 ± 0.01 (*p* < 0.0001), which did not differ from 1 (95% confidence limits; 0.97 < β < 1.02). When comparing the two adjusted models, RFI_bf1_ and RFI_bf2_ had strong Pearson and Spearman correlation coefficients at 0.95 (*p* < 0.0001) and 0.95 (*p* < 0.0001), respectively. Linear regression of RFI_bf1_ on RFI_bf2_ revealed a regression coefficient of 1.05 ± 0.05 (*p* < 0.0001), which did not differ from 1 (95% confidence limits; 0.96 < β < 1.14). Kappa coefficients between RFI_1_ and RFI_2_ and between RFI_bf1_ and RFI_bf2_ were 0.85 (95% confidence limits; 0.72 < β < 0.98) and 0.77 (95% confidence limits; 0.61 < β < 0.93), respectively, indicating a high level of agreement beyond chance. Kappa coefficients between RFI_2_ and RFI_bf2_ and between RFI_1_ and RFI_bf1_ were 0.69 (95% confidence limits; 0.52 < β < 0.87) and 0.62 (95% confidence limits; 0.43 < β < 0.81), respectively, indicating a fair to good level of agreement beyond chance.

### 3.4. Examination of Age at First Calving Relative to RFI Model in Brangus Heifers with RFI Phenotypes

There were no significant differences among RFI classification for age at first calving using Model 1, Model 2, or Model 4. Model 3 least squares means for medium and high RFI_2_ classified heifers were significantly different (*p* = 0.0422), where high RFI classified heifers calved 32 d earlier than medium RFI classified heifers. High RFI classified heifers were the youngest at first calving in all four models. Calving age and off-test weight had Pearson and Spearman correlation coefficients of −0.45 (*p* = 0.0007) and −0.33 (*p* = 0.0162), respectively. Linear regression of calving age on off-test weight estimated a regression coefficient of −0.45 ± 0.13 (*p* < 0.0007).

## 4. Discussion

The use of hybrid breeds such as Brangus cattle that better match seedstock genetics to environmental conditions contributes towards improved agricultural production efficiency. The incorporation of novel, heritable selection criterion such as RFI into breeding programs likewise can increase feed efficiency in progeny and this approach is being increasingly adopted in countries such as the US, Canada, and Australia [38]. Herein we report data that validates the RFI model for use in Brangus cattle and indicates on-test duration can be shortened to as little as 56-d on trial. Furthermore, inclusion of ultrasound backfat had minimal impact on selection accuracy but its inclusion might serve to buffer against unintended changes in age at calving due to selection for RFI.

Results from this study indicate that predictions of DMI were equivalent when comparing DMI data collected from 56-d and a 70-d on-test durations supporting the hypothesis that a shorter trial duration is sufficient to accurately estimate DMI in Brangus cattle. Furthermore, a 42-d on-test duration was only associated with a minor loss in model accuracy as relatively few changes in animal rank by RFI were apparent. Results from this study agree with literature reports for *Bos taurus* cattle that indicate accurate measurement of DMI can be measured in less than 70-d [39,40,41,42]. One such study recommended shortening tests to 42-d for the collection of DMI data reporting Pearson and Spearman correlation coefficients for a 42-d test of 0.97, which are equivalent to our findings for a 56-d test period and slightly greater than our findings for a 42-d test period [42]. Those authors further reported a regression coefficient of 0.99 (*p* < 0.0001) and a *R*^2^ of 0.97 for a 42-d test, which are higher than those in this study for either a 42-d or 56-d trial [42]. Another such study recommended a 35-d test for daily feed intake and reported a phenotypic correlation of 0.87 between a 35-d and 119-d test [24]. A similar Spearman correlation coefficient of 0.88 was reported in this study for a 28-d test. In another study, changes of phenotypic residual variances for DMI stabilized after 35 d on test and Pearson and Spearman correlations between a 35-d test and a 91-d test reached 0.93 [41]. When examining *Bos taurus* and *Bos indicus* cattle, another group reported the residual variance for DMI stabilized at 56-d and a 56-d test was appropriate to measure DMI [41]. Collectively these studies agree with our results and support the adoption of shorter on-test intervals for estimating RFI in Brangus cattle.

Another essential component of the RFI model involves accurate estimation of ADG. Our data indicate that a test duration of 56-d was equally predictive as the standard 70-d duration since regression coefficients were the same for both measures of ADG examined in this study. While correlations were slightly stronger for ADG_1_, this only indicates ADG_1_ for 56 d had a slightly stronger relationship with ADG_1_ for 70-d. Additionally, correlation coefficients between two measures that are progressively similar are not reliable indicators of the most accurate method due to autocorrelation. By these values alone, a definitive measure for calculating ADG cannot be determined when predicting expected feed intake. However, these results support those of other studies, which suggest estimating ADG rather than DMI is the limiting factor concerning test duration when calculating RFI [39,40,41,42]. A recent study utilizing *Bos taurus* bulls, steers, and heifers recommended shortening trial length from 70-d to 56-d for the collection of ADG data [42]. That study reported an *R*^2^ value, Pearson correlation coefficient, and Spearman correlation coefficient for a 56-d test of 0.95, 0.95, and 0.94, respectively, which are greater than our findings for a 56-d trial. Those authors further reported a regression coefficient of 0.80 (*p* < 0.005), which is slightly lower than what is reported in this study [42]. Another study recommended a 70-d test length for RFI trials and reported a phenotypic correlation of 0.85 between a 70-d and 119-d test [39]. Consistent with that study, a similar Spearman correlation coefficient of 0.86 was observed in this study for a 42-d test using ADG_1_ values and for a 56-d test using ADG_2_ values. Using the grow safe system, changes of phenotypic residual variances for ADG continued to fluctuate throughout a 91-d test period, indicating ADG requires a longer testing period and more measurements are needed to obtain an accurate determination of test duration [41]. However, in that study, Pearson and Spearman correlations between a 63-d test and a 91-d test were 0.90 and 0.87, respectively, and a 63-d test was sufficient for measuring ADG [41]. Another study examining on test duration in Angus, Hereford, Simmental, and Afrikaner genetics reported the residual variance for ADG stabilized after 42-d, and a test between 42-d and 56-d is sufficient for measuring ADG when linear regression is used to model weight vs. time [40]. These results also agree with the findings of our study where 56-d on-test is a viable alternative to the standard 70-d duration regarding the estimate of ADG in Brangus heifers.

While reducing RFI test duration is dependent on the accuracy of ADG measurements, it may be possible to overcome this limitation by adopting strategies that increase ADG estimations. Currently, the Beef Improvement Federation recommends a 21-d pre-test acclimation period followed by a 70-d performance test that includes daily DMI measurements and assessment of ADG with biweekly body weight determinations [18]. Collecting body weight data at more frequent intervals may facilitate shorter on-test intervals by increasing the accurate estimate of individual animal ADG. Weighing cattle weekly facilitated reduction in test durations for measuring ADG to 63-d [41]. Another study reported that test duration for ADG measurements could be shortened to as little as 42-d when cattle were weighed weekly and then fasted for at least 12 h before weighing [40]. While allowing test-duration to be shortened by providing a more accurate measure of body weight in part by reducing variation due to gut-fill, pre-weight fasting also disrupts normal feeding patterns, which could impact RFI accuracy by confounding estimation of DMI. In contrast, another study reported that when using a longer weighing frequency, a 70-d test is necessary to measure ADG [24]. Alternatively, recording live weights at periodic intervals during the test period and calculating rate of gain by regression may enhance the accuracy of measured rate of gain and allow for a slightly shorter test period [18]. While these strategies were not evaluated in the current study, their potential use for performance testing in Brangus cattle should be further investigated.

Several studies indicate RFI is weakly correlated with measures of body composition, specifically backfat thickness; therefore, the long-term selection for RFI might affect body composition as more efficient cattle tend to be leaner [17,23,24,25,26,27,28,29]. Thus, adjusting the RFI model for ultrasound backfat thickness has been proposed as a strategy to overcome this potential limitation though collecting such data adds significant expense to performance testing. In the current study, adjusting RFI for backfat only explained an additional 2% variation in DMI suggesting adjusting for backfat thickness had little impact. These results are consistent with others as a similar increase in model *R*^2^ (3%) was reported when including gain in ultrasound backfat thickness in the linear regression predicting DMI in Angus bulls [29]. A study in Brangus heifers revealed a slightly higher model *R*^2^ of 0.555 when predicting DMI from ADG and MMWT, but the increase of *R*^2^ (4.2%) when including gain in ultrasound backfat thickness was like the increase in *R*^2^ seen in the current study using the calving data subset [17]. Two additional studies reported smaller increases in *R*^2^ of 1.4% and 1.8%, respectively, when adjusting RFI for body composition [25,27]. In contrast, a study utilizing crossbred steers reported correlations between RFI and ultrasound backfat thickness were stronger, suggesting that selection for RFI might result in selection for leaner animals [28]. However, in an earlier study using *Bos taurus* cattle, small phenotypic correlations between RFI and ultrasound backfat with low RFI cattle having reduced ultrasound backfat thickness were reported, in comparison to high RFI cattle [26].

In this regard, one study reported RFI adjusted for ADG, MMWT, and ultrasound backfat accounted for 66.1% and 75.3% of the variation in expected DMI for Angus and Charolais steers, respectively [30]. While the addition of UBF into the RFI model only accounted for an additional 0.5% variation in DMI for Angus steers, there was a much larger effect on Charolais steers and accounted for an additional 2.3% variation in DMI. Those authors concluded that a larger impact was made in Charolais steers because they tend to mature later than Angus cattle [30]. Larger framed animals that tend to mature later are more efficient relative to smaller framed, earlier maturing cattle when compared at similar weights. Taken together, results from the current study agree with literature reports that there were no significant differences between heifers based on RFI classification when adjusted for ultrasound backfat thickness. However, differences in ultrasound backfat thickness between heifers based on RFI classification suggest there is a weak relationship between the two. While this may not affect the accuracy of selection in seedstock animals, inclusion of ultrasound measures of body composition on the computation of RFI may be useful to reduce the impact of selection on carcass quality of steer progeny during finishing.

Interestingly, our results suggest that as off-test bodyweight increases, age at first calving decreases. Indeed, when RFI was not adjusted for UBF, high RFI classified heifers were the heaviest for off-test bodyweight. This is consistent with current research suggesting high RFI females calve earlier in the calving season because their more efficient counterparts tend to have a delay in pregnancy most likely attributed to a delay in first estrus [33,34,35,43]. Maximizing reproductive performance is essential for the sustainability of beef production systems. Therefore, genetic selection for traits with potential negative effects on reproduction is not recommended. Feed intake trials are conducted post-weaning prior to selection decisions being made. Because there is a large variation in age at puberty, *Bos taurus* and *Bos taurus* influenced cattle tend to be at different stages of sexual development during this time and differences in physiological age may confound RFI classification. *Bos indicus* cattle are generally older when reaching puberty compared to *Bos taurus* breeds, which could confound any effects of delayed breeding associated with selection for RFI. Consequentially, RFI testing tends to favor later maturing animals that do not have increased energy demands associated with sexual development and activity; therefore, prepubertal animals have lower DMI than those undergoing puberty [43]. Results from this study suggest RFI should be adjusted for ultrasound backfat thickness as adjusted RFI models in the present study were independent of age at first calving. While there were not any significant differences between age at calving and UBF, the adjusted RFI models suggest underlying processes associated with body composition may affect reproductive performance and adjusting for backfat thickness eliminates differences in physiological age that may exist in heifers while on test.

## 5. Conclusions

This study supports the conclusion that performance test duration for measuring feed intake can be reduced to 56-d and this recently adopted recommendation by the Beef Improvement Federation Guidelines based upon *Bos taurus* cattle holds for *Bos indicus* influenced cattle. Shorting on-test duration by 14 days confers significant economic benefit for seedstock producers who incorporate RFI as a selection criterion and potentially allows more animals to be evaluated annually. Inclusion of ultrasound backfat in the RFI model had minimal impact on the accuracy of selection in Brangus cattle. Therefore, ultrasound measures of body composition are unnecessary for genetic improvement of RFI in seedstock animals. However, growth and performance records reveal inclusion of ultrasound backfat thickness into RFI model may prevent potential indirect effects that long-term selection for RFI might have on carcass quality of progeny. Additionally, high RFI classified heifers were significantly younger at first calving when RFI was not adjusted for ultrasound backfat thickness. These differences were no longer significant when the RFI model was adjusted for ultrasound backfat thickness. Therefore, adjusting RFI for ultrasound backfat allows confounding differences in heifer development between animals on-test to be better controlled. However, further research is warranted to thoroughly investigate whether the added costs associated with the collection of ultrasound data are worth the upfront investment.

## Figures and Tables

**Table 1:** Formation of contemporary group by on-test date and source of farm.

Contemporary Group	Date	Farm	Number of Heifers
1	June 2014 to September 2014	1	20
2	June 2015 to August 2015	1	46
3	June 2014 to September 2014	2	22
4	August 2015 to October 2015	2	23
5	September 2015 to December 2015	1	22
6	December 2014 to March 2015	1	12
7	December 2014 to March 2015	2	41
Total			186

**Table 2:** Ingredient composition and nutritional value of diet fed to Brangus heifers.

Item	Value, %
**Dietary composition, (as fed)**	
Cracked corn	13.75
Soyhull pellets	20
Dried distillers grain	5
Corn gluten pellets	22.5
Cottonseed hull pellets	15
Alfalfa meal	5
Mineral	2.5
Potassium chloride	0.15
Supplement	0.1
Cottonseed hulls	10
Molasses	6
**Chemical composition, (DM basis)**	
CP, %	13.4
NDF, %	44.1
ME, Mcal/kg	2.47
NE_m_, Mcal/kg	0.7
NEg, Mcal/kg	0.42

**Table 3:** Means (±SD) for performance traits by contemporary group based on a 70-d test.

Group	N	Initial BW, kg	Final BW, kg	DMI, kg/d	ADG_1_ ^1^, kg/d	ADG_2_ ^2^, kg/d	MMWT_1_ ^3^, kg	MMWT_2_ ^4^, kg
1	20	301.87 ± 32.68	394.88 ± 34.21	9.58 ± 0.78	1.30 ± 0.18	1.32 ± 0.19	66.26 ± 4.68	66.12 ± 4.64
2	46	301.70 ± 31.98	392.56 ± 34.92	10.04 ± 1.18	1.29 ± 0.13	1.30 ± 0.13	65.96 ± 4.74	65.94 ± 4.76
3	22	278.24 ± 24.97	362.57 ± 30.49	8.40 ± 0.97	1.19 ± 0.15	1.21 ± 0.15	62.19 ± 3.91	62.10 ± 4.00
4	23	292.08 ± 29.62	397.01 ± 40.09	9.76 ± 1.50	1.50 ± 0.23	1.50 ± 0.23	65.61 ± 4.93	65.57 ± 4.92
5	22	323.72 ± 33.85	436.59 ± 42.27	10.96 ± 1.54	1.61 ± 0.21	1.58 ± 0.23	70.61 ± 5.34	70.75 ± 5.24
6	12	325.38 ± 28.98	430.20 ± 33.89	10.61 ± 1.59	1.55 ± 0.27	1.57 ± 0.28	70.03 ± 4.24	69.93 ± 4.31
7	41	299.49 ± 25.44	397.57 ± 35.30	9.57 ± 1.56	1.46 ± 0.24	1.40 ± 0.23	65.86 ± 4.22	66.15 ± 4.22

^1^ ADG_1_ is calculated by the linear regression of BW on days on test. ^2^ ADG_2_ is calculated by (final BW − initial BW)/days on test. ^3^ MMWT_1_ is calculated by (final BW − (0.5 × days on test × ADG_1_))^0.75^. ^4^ MMWT_2_ is calculated by (final BW − (0.5 × days on test × ADG_2_))^0.75^.

**Table 4:** Least squares means ± SEM for growth and performance traits of heifers by residual feed intake (RFI_1_ ^1^) (kg/d).

Trait	N	RFI_1_ ^1^			*p*-Value
		Low (*n* = 29)	Medium (*n* = 130)	High (*n* = 27)	
RFI_1_ ^1^, kg/d	186	−1.44 ± 0.12 ^a^	−0.08 ± 0.07 ^b^	1.43 ± 0.13 ^c^	0.0001
Initial BW, kg	186	295.63 ± 6.26	299.91 ± 3.69	298.03 ± 6.75	0.8241
Final BW, kg	186	391.72 ± 7.59	396.48 ± 4.48	393.07 ± 8.19	0.8211
DMI, kg/d	186	8.27 ± 0.21 ^a^	9.61 ± 0.12 ^b^	11.01 ± 0.23 ^c^	0.0001
ADG_1_ ^2^, kg/d	186	1.40 ± 0.05	1.39 ± 0.03	1.39 ± 0.05	0.9607
MMWT_1_ ^3^, kg	186	65.30 ± 0.97	66.06 ± 0.57	65.53 ± 1.04	0.7372
UBF ^4^, mm	175	6.69 ± 0.46	7.85 ± 0.29	7.80 ± 0.53	0.1001

^1^ RFI_1_ is adjusted for ADG calculated by linear regression (ADG_1_) and metabolic midweight (MMWT_1_). ^2^ ADG_1_ is calculated by the linear regression of BW on days on test. ^3^ MMWT_1_ is calculated by (final BW − (0.5 × days on test × ADG_1_))^0.75^. ^4^ 70 d ultrasound backfat thickness. ^a–c^ Least squares mean within a row are significantly different (*p* < 0.0001).

**Table 5:** Regression coefficients, *R*^2^, and correlations for average daily DMI (kg/d) over 70 d regressed on shorter durations within the 70-d test.

70-d Values Regressed on	DMI, kg/d				
	Regression Coefficient ^1^	SE	*R* ^2^	Pearson ^2^	Spearman ^3^
0 to 14 d	0.80	0.04	0.69	0.83	0.84
0 to 28 d	0.87	0.03	0.79	0.89	0.88
0 to 42 d	0.94	0.02	0.89	0.95	0.94
0 to 56 d	0.96	0.02	0.94	0.97	0.97

^1^ All regression coefficients were statistically different from 0 where *p* < 0.0001. ^2^ All Pearson correlations were statistically different from 0 where *p* < 0.0001. ^3^ All Spearman correlations were statistically different from 0 where *p* < 0.0001.

**Table 6:** Regression coefficients, *R*^2^, and correlations for ADG_1_ ^1^ (kg/d) and ADG_2_^2^ (kg/d) over 70-d regressed on shorter durations within the 70-d test.

70-d Values Regressed on	ADG_1_ ^1^, kg/d					ADG_2_ ^2^, kg/d				
	Regression Coefficient ^3^	SE	*R* ^2^	Pearson ^4^	Spearman ^5^	Regression Coefficient ^6^	SE	*R* ^2^	Pearson ^7^	Spearman ^8^
0 to 14 d	0.12	0.04	0.04	0.21	0.29	0.08	0.04	0.02	0.16	0.22
0 to 28 d	0.54	0.04	0.50	0.71	0.73	0.52	0.03	0.55	0.74	0.77
0 to 42 d	0.70	0.03	0.76	0.87	0.86	0.63	0.04	0.61	0.78	0.82
0 to 56 d	0.84	0.02	0.86	0.93	0.90	0.84	0.04	0.74	0.86	0.86

^1^ ADG_1_ is calculated by the linear regression of BW on days on test. ^2^ ADG_2_ is calculated by (final BW − initial BW)/days on test. ^3^ All regression coefficients were statistically different from 0 where *p* < 0.005. ^4^ All Pearson correlations were statistically different from 0 where *p* < 0.005. ^5^ All Spearman correlations were statistically different from 0 where *p* < 0.0001. ^6^ All regression coefficients were statistically different from 0 where *p* < 0.05. ^7^ All Pearson coefficients were statistically different from 0 where *p* < 0.05. ^8^ All Spearman coefficients were statistically different from 0 where *p* < 0.005.

**Table 7:** Regression coefficients, *R*^2^, and correlations for ADG_1_ ^1^ (kg/d) and ADG_2_^2^ (kg/d) over 70-d regressed on shorter durations within the 70-d test.

70-d Values Regressed on	RFI_1_ ^1^, kg/d					RFI_2_ ^2^, kg/d				
	Regression Coefficient ^3^	SE	*R* ^2^	Pearson ^4^	Spearman ^5^	Regression Coefficient	SE	*R* ^2^	Pearson	Spearman
0 to 14 d	0.56	0.05	0.43	0.65	0.68	0.56	0.05	0.43	0.65	0.68
0 to 28 d	0.76	0.04	0.67	0.82	0.81	0.76	0.04	0.67	0.82	0.81
0 to 42 d	0.87	0.03	0.80	0.90	0.89	0.85	0.03	0.78	0.88	0.88
0 to 56 d	0.93	0.02	0.90	0.95	0.95	0.91	0.03	0.88	0.94	0.93

^1^ RFI_1_ is adjusted for ADG_1_ and MMWT_1_, where ADG_1_ is calculated by linear regression and MMWT_1_ is calculated by (final BW − (0.5 × days on test × ADG_1_))^0.75^. ^2^ RFI_2_ is adjusted for ADG_2_ and MMWT_2_, where ADG_2_ is calculated by (final BW − initial BW)/days on test and MMWT_2_ is calculated by (final BW − (0.5 × days on test × ADG_2_))^0.75^. ^3^ All regression coefficients were statistically significant where *p* < 0.0001. ^4^ All Pearson correlations were statistically significant where *p* < 0.0001. ^5^ All Spearman correlations were statistically significant where *p* < 0.0001.

## Data Availability

The data presented in this study are available on request from the corresponding author.
